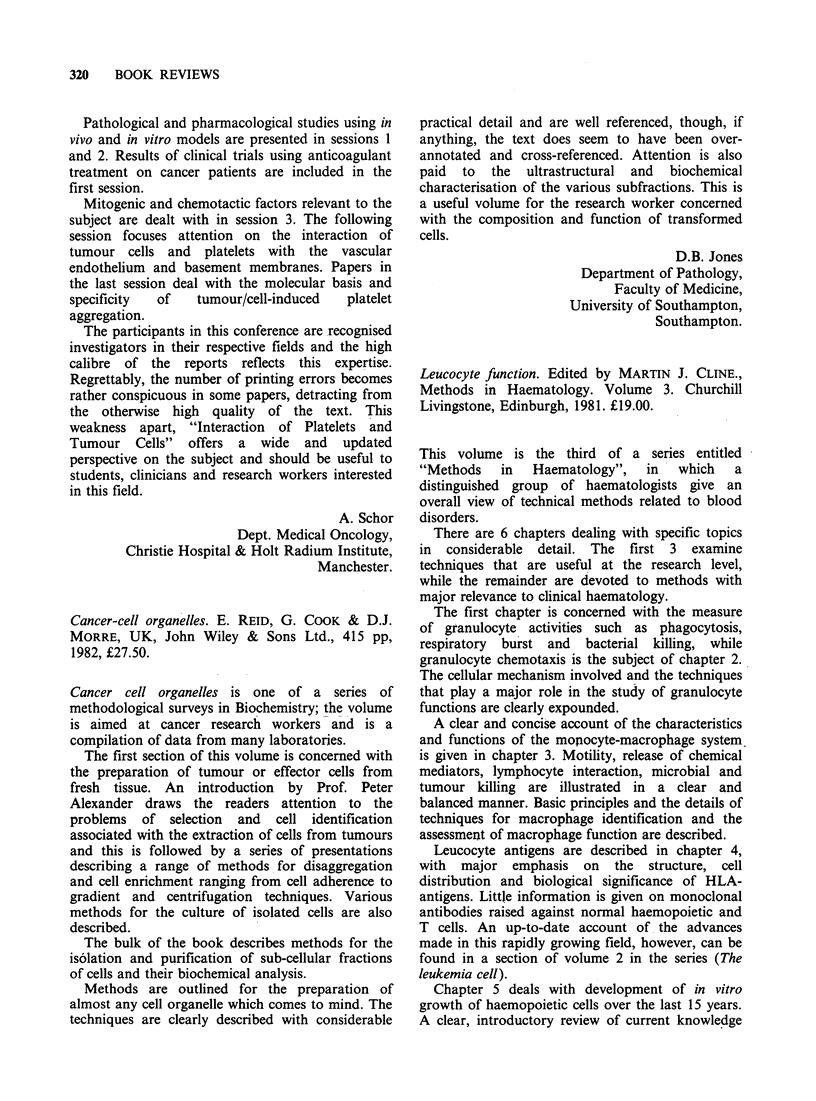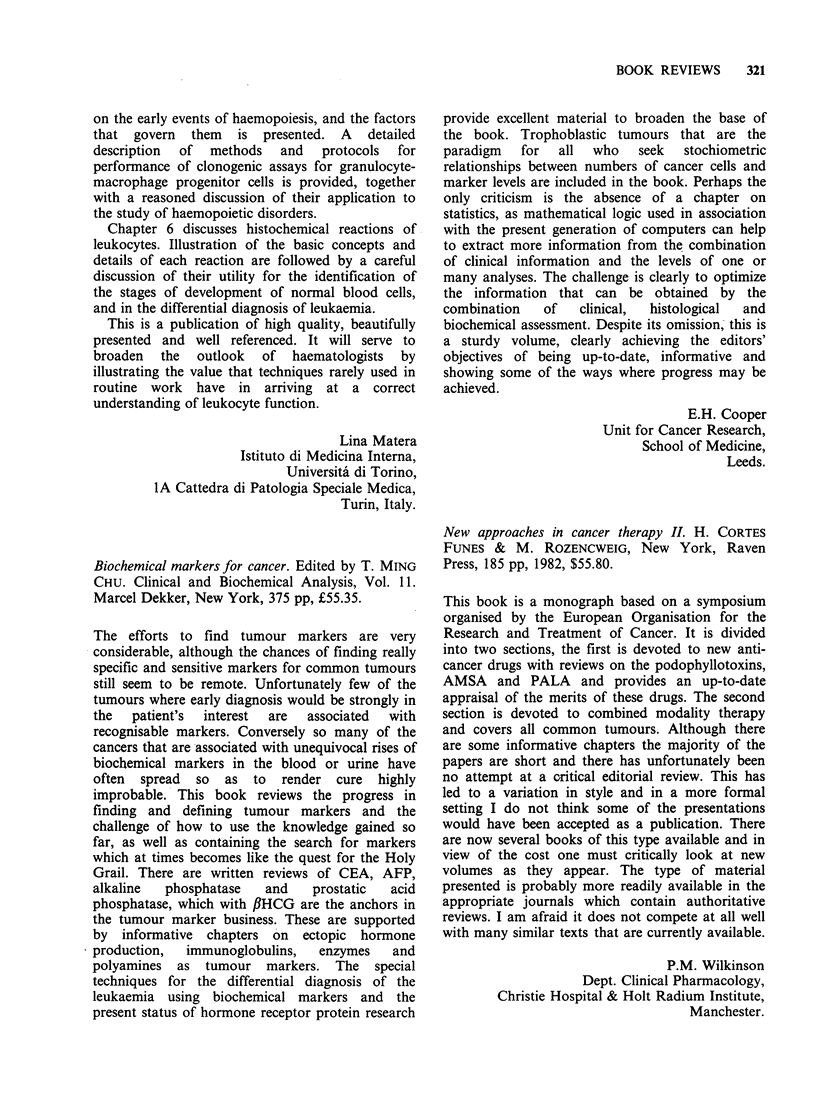# Leucocyte function

**Published:** 1983-02

**Authors:** Lina Matera


*Leucocyte function*. Edited by MARTIN J. CLINE., Methods in Haematology. Volume 3. Churchill Livingstone, Edinburgh, 1981. £19.00.

This volume is the third of a series entitled "Methods in Haematology", in which a distinguished group of haematologists give an overall view of technical methods related to blood disorders.

There are 6 chapters dealing with specific topics in considerable detail. The first 3 examine techniques that are useful at the research level, while the remainder are devoted to methods with major relevance to clinical haematology.

The first chapter is concerned with the measure of granulocyte activities such as phagocytosis, respiratory burst and bacterial killing, while granulocyte chemotaxis is the subject of chapter 2. The cellular mechanism involved and the techniques that play a major role in the study of granulocyte functions are clearly expounded.

A clear and concise account of the characteristics and functions of the monocyte-macrophage system is given in chapter 3. Motility, release of chemical mediators, lymphocyte interaction, microbial and tumour killing are illustrated in a clear and balanced manner. Basic principles and the details of techniques for macrophage identification and the assessment of macrophage function are described.

Leucocyte antigens are described in chapter 4, with major emphasis on the structure, cell distribution and biological significance of HLA-antigens. Little information is given on monoclonal antibodies raised against normal haemopoietic and T cells. An up-to-date account of the advances made in this rapidly growing field, however, can be found in a section of volume 2 in the series (*The leukemia cell*).

Chapter 5 deals with development of *in vitro* growth of haemopoietic cells over the last 15 years. A clear, introductory review of current knowledge on the early events of haemopoiesis, and the factors that govern them is presented. A detailed description of methods and protocols for performance of clonogenic assays for granulocyte-macrophage progenitor cells is provided, together with a reasoned discussion of their application to the study of haemopoietic disorders.

Chapter 6 discusses histochemical reactions of leukocytes. Illustration of the basic concepts and details of each reaction are followed by a careful discussion of their utility for the identification of the stages of development of normal blood cells, and in the differential diagnosis of leukaemia.

This is a publication of high quality, beautifully presented and well referenced. It will serve to broaden the outlook of haematologists by illustrating the value that techniques rarely used in routine work have in arriving at a correct understanding of leukocyte function.

Lina Matera
Istituto di Medicina Interna,
Universitá di Torino,
1A Cattedra di Patologia Speciale Medica,
Turin, Italy.